# Supplementary material for: Spatial distribution and predictive factors of antenatal care in Burundi: A spatial and multilevel baseline analysis for the third burundian demographic and health survey
Source: PLoS One. 2023 Jan 17;18(1):e0272897. doi: 10.1371/journal.pone.0272897 (PMC9844929; doi:10.1371/journal.pone.0272897)
Supplement: Attachment — Submitted filename: Review comments to author.pdf [file pone.0272897.s001.pdf]

Title:- Spatial distribution and predictive factors of antenatal care in Burundi: a multilevel analysis and spatial baseline for the third Burundian demographic health survey

## Accept with Major revision

General comments

- ✓ The entire section of the manuscript contained missing/incomplete words.

Abstract

- ✓ The methods used to present findings (table, graphs, text, etc.) should be indicated in the abstract section.
- ✓ The authors should describe the analysis techniques used to determine the determinant factors for ANC.
- ✓ The authors should describe the method used to report the effect size as well as the level of significance in the abstract section.

Background

- ✓ The authors should revise the background section

Methods

- ✓ In the title you have stated the analysis as a multilevel analysis and spatial baseline for the third Burundian demographic health survey, but I have not seen any evidence in the method as well as in the result section that you have done a multilevel analysis, what was your intention to say a multilevel analysis?
- ✓ In the method section, study variables part, the authors didn’t state about their dependent variable. Also the independent variables from previous studies should have to be merged and presented in a single paragraph.

Results

- ✓ In the result section, on the sub section of Predictive factors of antenatal care, the authors have stated ANC with a medical doctor as a dependent variable. As per the DHS guide, women receiving antenatal care from a skilled provider for the most recent birth will be considered as having antenatal care. What is the reason behind choosing only ANC with a medical doctor?
- ✓ In the result section, on the sub section of Predictive factors of antenatal care, replace the word univariate analysis by univariable analysis. Also replace the word multivariate analysis by multivariable analysis.
- ✓ In the result section, education level and the delivery place were reported as a significant predictor of ANC. unless it is a previous delivery, how place of delivery affects ANC utilization?
- ✓ Better to present the spatial distribution in a scientific way and report important results. For example, rather than doing the “Spatial distribution of health centers in Burundi” give emphasis for your outcome variable
