# Supplementary material for: Spatial distribution and predictive factors of antenatal care in Burundi: A spatial and multilevel baseline analysis for the third burundian demographic and health survey
Source: PLoS One. 2023 Jan 17;18(1):e0272897. doi: 10.1371/journal.pone.0272897 (PMC9844929; doi:10.1371/journal.pone.0272897)
Supplement: Attachment — Submitted filename: My comments.docx [file pone.0272897.s002.docx]

Manuscript Title: Spatial Distribution and Predictive Factors of Antenatal Care in Burundi: A Spatial and Multilevel Baseline Analysis for the Third Burundian Demographic and Health Survey

Manuscript id: PONE-D-22-21281R1

General comment

I saw the manuscript and previous comments are addressed. It is also prepared as per the PLOS One guideline.
